# Lightweight Optimization of a Steering Knuckle for Additive Manufacturing: Experimental Characterization and Numerical Assessment

**DOI:** 10.3390/ma19214437

**Published:** 2026-10-22

**Authors:** Carlos Soto Encinas, Carlos Garza, Miguel F. Delgado-Pamanes, Johana Gamez, Roberto Cabriales, Patricia C. Zambrano-Robledo, Luis A. Reyes-Osorio

**Affiliations:** 1 Universidad Autónoma de Nuevo León, Facultad de Ingeniería Mecánica y Eléctrica, Centro de Investigación e Innovación en Ingeniería Aeronáutica, San Nicolás de los Garza 66600, N.L., Mexico; carlos.sotoe@uanl.edu.mx (C.S.E.); carlos.garzardrg@uanl.edu.mx (C.G.); roberto.cabrialesgm@uanl.edu.mx (R.C.); 2 Instituto Politécnico Nacional, Unidad Profesional Interdisciplinaria de Ingeniería Campus Zacatecas, Zacatecas 98160, Mexico; mfdelgadop@ipn.mx

**Keywords:** laser-based powder bed fusion of metals, AlSi10Mg, SiC particles, process simulation, finite element analysis, steering knuckle, structural optimization

## Abstract

The increasing demand for lightweight components has intensified interest in additively manufactured aluminum alloys and structural optimization strategies. This study investigates the lightweight optimization of a Formula SAE steering knuckle intended for laser-based powder bed fusion of metals (PBF-LB/M) through an integrated methodology combining experimental material characterization, manufacturing-process simulation, finite element analysis, and geometry optimization. PBF-LB/M-fabricated AlSi10Mg and AlSi10Mg/SiC specimens were experimentally characterized through tensile and comparative high-cycle fatigue testing, while Charpy impact testing and fractographic analysis were performed on the AlSi10Mg/SiC composite. The experimental results indicated that SiC reinforcement did not improve mechanical performance under the investigated processing conditions, highlighting the potential influence of reinforcement dispersion, interfacial integrity, and process-induced heterogeneities. Component-level numerical investigations were conducted exclusively for AlSi10Mg. The PBF-LB/M manufacturing process was simulated using Simufact Additive to assess residual stresses and geometric distortion following support and base-plate release. Subsequently, structural finite element analysis under representative Formula SAE loading conditions provided the basis for geometry optimization. The optimized steering knuckle achieved a 45.1% mass reduction, from 1.13 to 0.62 kg, with a maximum von Mises stress of approximately 89 MPa and a minimum safety factor of 2.52 under the investigated static loading conditions. These findings demonstrate the potential of combining experimental material characterization with process simulation and structural optimization for lightweight AlSi10Mg components. The AlSi10Mg/SiC results are limited to specimen-level characterization, and its component-level structural performance remains to be investigated.

## 1. Introduction

The transition toward sustainable and energy-efficient mobility has intensified the demand for lightweight structural components capable of maintaining high mechanical performance under increasingly complex service conditions [1]. In automotive systems, weight reduction can contribute to improved fuel efficiency, extended electric-vehicle range, and reduced energy consumption, making lightweight design an important engineering objective. However, achieving substantial mass reduction while preserving structural integrity remains challenging, particularly for safety-critical components subjected to multiaxial, dynamic, and cyclic loading conditions [2].

Among such components, the steering knuckle represents a structurally demanding element because it serves as the mechanical interface between the suspension, braking, and steering systems. During operation, it is subjected to combinations of vertical, lateral, and longitudinal forces, together with transient and cyclic loads associated with acceleration, braking, cornering, and road irregularities [3]. These demanding loading conditions, combined with the need to minimize mass, make the steering knuckle a suitable component for evaluating lightweight materials and structural optimization strategies.

The development of lightweight structural components has been closely linked to advances in manufacturing technologies. In this context, laser-based powder bed fusion of metals (PBF-LB/M) has emerged as a promising approach for producing geometrically complex metallic components through layer-by-layer rapid solidification [4]. Compared with conventional manufacturing processes, PBF-LB/M provides considerable design freedom and facilitates the fabrication of geometries resulting from topology optimization. Nevertheless, the process involves steep thermal gradients, rapid cooling rates, and repeated thermal cycling, which can generate microstructural heterogeneity, residual stresses, distortion, and process-induced defects such as porosity and lack of fusion. These features can significantly influence the mechanical response and structural reliability of PBF-LB/M-fabricated components [5,6,7].

AlSi10Mg alloy has become a widely investigated material for PBF-LB/M because of its favorable processability, good mechanical performance and low density. Its near-eutectic composition provides favorable melt behavior and relatively low susceptibility to solidification cracking, facilitating the production of components under appropriate processing conditions. However, the mechanical response of PBF-LB/M-fabricated AlSi10Mg remains strongly dependent on processing parameters, defect populations, and post-processing treatments. Fatigue and impact performance are particularly sensitive to these factors, which may limit the reliability of the material in components subjected to repeated or dynamic loading [8,9].

The incorporation of reinforcement into PBF-LB/M-processed Al alloys has therefore been investigated as a potential strategy for improving selected mechanical properties through microstructural refinement and local strengthening. Metal matrix composites reinforced with silicon carbide are of particular interest because they combine the low density and processability of Al alloys with the high stiffness, hardness, and thermal stability of SiC [10,11,12]. Potential strengthening mechanisms include load transfer, Orowan strengthening, grain refinement through heterogeneous nucleation, and changes in interfacial phase formation. However, the effectiveness of these mechanisms depends strongly on reinforcement size, morphology, distribution and processing conditions.

Previous research demonstrates that the beneficial effect of SiC reinforcement is not universal but results from a balance between reinforcement-driven strengthening and defect-driven degradation. Xue et al. [13] reported densification levels above 98% in PBF-LB/M-fabricated SiC/AlSi10Mg composites under optimized processing conditions, while inadequate parameter selection and reinforcement distribution adversely affected mechanical performance. Crack initiation associated with pores and fractured SiC particles further demonstrated that reinforcement could act as a local stress concentrator. Other studies have shown that reducing reinforcement size and improving particle distribution can enhance mechanical properties, emphasizing the importance of controlling reinforcement characteristics and processing conditions [14].

More recent investigations showed that particle morphology influences powder behavior, densification, and mechanical performance, with spherical SiC particles promoting improved flowability and more homogeneous reinforcement distribution [15]. Song et al. [16] determined that increasing SiC content enhanced hardness and strength but progressively reduced fracture resistance.

For steering knuckle applications, material selection must consider adequate strength, ductility, and stiffness to withstand the static and dynamic loading conditions encountered during Formula SAE competition. Because the component experiences repeated and variable loads, fatigue performance is particularly important for structural durability. In PBF-LB/M-processed AlSi10Mg, fatigue behavior is highly sensitive to process-induced defects, including pores, lack-of-fusion regions, and surface irregularities, which can serve as preferential sites for crack initiation. Reinforcement phases may modify cyclic performance through microstructural refinement and strengthening mechanisms. Similar considerations apply to impact behavior, as process-induced defects and reinforcement-related heterogeneities can reduce the ability of the material to accommodate localized deformation and may promote brittle fracture.

Despite extensive research on the mechanical behavior of PBF-LB/M Al alloys and the structural optimization of automotive components, these aspects are frequently investigated independently. Material characterization studies typically focus on specimen-level mechanical performance and process–microstructure relationships, whereas steering-knuckle optimization studies commonly rely on nominal or literature-based material properties without explicitly considering the experimentally measured response of additively manufactured materials or manufacturing-induced effects. Consequently, an integrated framework connecting PBF-LB/M material characterization, manufacturing-process simulation, component-level structural analysis, and lightweight optimization remains valuable for assessing the feasibility of transferring additively manufactured material systems to structurally demanding applications.

Accordingly, the present study investigates the lightweight design and structural performance of a Formula SAE steering knuckle intended for PBF-LB/M fabrication. The proposed methodology integrates experimental material characterization, manufacturing-process simulation, finite element analysis, and topology optimization. AlSi10Mg and AlSi10Mg/SiC specimens were evaluated through tensile and comparative high-cycle fatigue testing, while impact testing and fractographic analysis were performed exclusively on the composite.

The experimental investigation compares the mechanical behavior of both materials at the specimen level. In contrast, manufacturing-process simulation using Simufact Additive, component-level structural analysis, and topology optimization were conducted exclusively for monolithic AlSi10Mg. This distinction establishes the scope of the study, demonstrating an integrated material-to-component design framework while identifying the process-dependent limitations of SiC reinforcement. The component-level structural performance of AlSi10Mg/SiC is not considered in the present investigation.

## 2. Materials and Methods

### 2.1. Materials, Powder Preparation, and PBF-LB/M Processing

The specimens were fabricated using an SLM 280 HL system (SLM Solutions^®^, Lübeck, Germany). The machine is equipped with a continuous-wave ytterbium fiber laser (IPG Photonics Corporation, Marlborough, MA, USA) with a wavelength of 1064 nm, a nominal power of 400 W, and a laser spot diameter of 70 μm. During fabrication, the build platform was preheated to 200 °C, while the processing chamber was maintained under a controlled argon atmosphere to minimize oxidation. The AlSi10Mg powder supplied by Carpenter Additive presented a nominal composition (in wt.%) of 9–11% Si, 0.2–0.45% Mg, 0.45% Mn, and 0.55% Fe, with Al as the balance element. The average particle size was 24.66 μm with spherical morphology. As the reinforcement phase, SiC nanoparticles supplied by US Research Nanomaterials Inc. were used. According to the manufacturer’s specifications, the particles had a purity of 99.5%, an average particle size of 65 nm, a polygonal morphology, and a true density of 3.21 g/cm^3^, with an apparent density of 0.05 g/cm^3^. For the preparation of the AlSi10Mg/SiC, the target reinforcement content was defined as 1.4 vol.% SiC.

#### 2.1.1. Feedstock Materials and Powder Mixing

Prior to fabrication, the feedstock powders underwent a preparation and conditioning procedure to improve powder homogeneity and promote SiC reinforcement dispersion within the AlSi10Mg matrix. Powder mixing, sieving, and handling were performed using a PSM 100 powder sieving system (SLM Solutions GmbH, Lübeck, Germany), as illustrated in Figure 1. The preparation procedure consisted of seven consecutive cycles of 20 min each under an argon-protected atmosphere. A low-energy mixing approach was employed to minimize changes in the original powder morphology while promoting a more homogeneous distribution of the SiC particles. No high-energy ball milling was performed.

Despite the repeated mixing and sieving cycles, complete dispersion of the SiC reinforcement could not be guaranteed, and residual particle agglomeration was observed. This limitation may have contributed to microstructural heterogeneity and influenced the subsequent mechanical response of the AlSi10Mg/SiC composite.

#### 2.1.2. Selection of PBF-LB/M Processing Parameters

A processing window was selected from previous studies reporting the fabrication of AlSi10Mg parts with high relative density (RD > 98%). This selection is illustrated in Figure 2, where the volumetric energy density is plotted as a function of the resulting relative density. Based on these data, a suitable processing interval ranging from 40 to 70 J/mm^3^ was identified.

For the fabrication of AlSi10Mg/SiC, an adjustment was required for the processing conditions due to the expected influence of reinforcement on laser absorption, melt pool dynamics, wetting behavior, and solidification response. Previous studies on PBF-LB/M-processed Al matrix composites have reported that the most favorable combinations of microstructural quality and mechanical performance are generally obtained within a range of approximately 100–150 J/mm^3^ [29,30,31]. The processing parameters used for the AlSi10Mg and AlSi10Mg/SiC are summarized in Table 1.

The processing parameters were selected according to the requirements of each feedstock. For AlSi10Mg, a hatch spacing of 0.13 mm resulted in a calculated volumetric energy density of approximately 57 J/mm^3^, within the processing window of 40–70 J/mm^3^ identified from the literature (Figure 2). For AlSi10Mg/SiC, the processing parameters were adjusted to account for the presence of reinforcement, resulting in a volumetric energy density of approximately 103 J/mm^3^.

#### 2.1.3. Relative Density Measurement

The relative density of the as-built materials was experimentally determined using the Archimedes method according to ASTM B962 [32], considering the presence of surface-connected porosity. Five measurements were performed for both AlSi10Mg and AlSi10Mg/SiC specimens to evaluate the actual densification achieved using the selected PBF-LB/M processing conditions.

### 2.2. Mechanical Characterization

Mechanical characterization was carried out on PBF-LB/M-fabricated AlSi10Mg and AlSi10Mg/SiC specimens. The experimental campaign included tensile, high-cycle fatigue, and Charpy impact testing. In addition, selected specimens were prepared for microstructural characterization to support the interpretation of the observed mechanical behavior. Specimens with different geometries were fabricated according to the requirements of each mechanical test. Tensile, high-cycle fatigue, and Charpy impact tests were conducted in accordance with ASTM E8/E8M-22 [33], ASTM E466-21 [34], and ASTM E23-24 [35], respectively. The geometries and dimensions of the specimens used for each test are presented in Figure 3.

The materials and conditions evaluated differed among the mechanical tests. High-cycle fatigue testing included AlSi10Mg and AlSi10Mg/SiC specimens, whereas Charpy impact testing was performed only on AlSi10Mg/SiC specimens.

#### 2.2.1. Tensile Testing

Uniaxial tensile tests were performed using a Shimadzu AGS-X universal testing machine with a load capacity of 10 kN. The tests were conducted at room temperature in accordance with ASTM E8/E8M [33], using a constant crosshead displacement rate of 1 mm/min until specimen failure. Tensile deformation was measured using Digital Image Correlation (DIC), with a speckle pattern applied to the specimen surface for optical deformation tracking. The geometry and dimensions of the tensile specimens are shown in Figure 3a.

#### 2.2.2. High-Cycle Fatigue Testing

High-cycle fatigue tests were conducted using a Satec 8801 fatigue testing machine in accordance with ASTM E466 [34]. The tests were performed under fully reversed axial loading conditions with a stress ratio of R = −1 and a loading frequency of 50 Hz. A maximum of 10^7^ cycles was established as the run-out criterion; specimens reaching this limit without failure were classified as runouts.

The fatigue testing was designed as a comparative high-cycle fatigue assessment at selected normalized stress levels rather than as a complete S–N fatigue characterization. The applied stress amplitudes were normalized with respect to the experimentally determined yield strength of each material, allowing their cyclic responses to be evaluated under comparable relative loading conditions. For the AlSi10Mg alloy, fatigue testing was conducted at a stress amplitude corresponding to 43% of its measured yield strength (224 MPa). For the AlSi10Mg/SiC, two normalized stress levels, corresponding to 43% and 50% of its measured yield strength, were evaluated. The additional stress level was included to assess the cyclic response of the reinforced material under more demanding loading conditions.

All fatigue specimens were tested in the as-built surface condition, without post-fabrication machining or other surface-finishing treatments. This condition was intentionally retained to evaluate fatigue performance in a representative PBF-LB/M surface state, in which surface roughness and near-surface defects may contribute to fatigue crack initiation. The geometry and dimensions of the fatigue specimens are shown in Figure 3b.

#### 2.2.3. Charpy Impact Testing

Charpy impact tests were conducted using a Tinius Olsen Model 84 (Tinius Olsen Testing Machine Co., Inc., Horsham, PA, USA) impact testing machine in accordance with ASTM E23-24 [35]. The tests were performed at room temperature (22.7 °C) to evaluate the impact energy absorption and fracture behavior of the AlSi10Mg/SiC samples under dynamic loading conditions. The Charpy test was included to complement the tensile and fatigue characterization by assessing the material response to sudden loading. The geometry and dimensions of the Charpy specimens are presented in Figure 3c.

### 2.3. Microstructural and Fractographic Characterization

A scanning electron microscope (SEM; VEGA3-SB, TESCAN, a.s., Brno, Czech Republic) operated at an accelerating voltage of 15 kV and a working distance of 15 mm was utilized for microstructure evaluation. Elemental analysis was performed using an EDAX energy-dispersive X-ray spectroscopy (EDS) detector. EDS analyses were used to examine the local elemental distribution and chemical characteristics of the matrix and reinforcement-containing regions. These observations were subsequently used to support the interpretation of the tensile, fatigue, and impact behavior of the PBF-LB/M-fabricated AlSi10Mg and AlSi10Mg/SiC materials. Fracture characteristics were considered to assess the AlSi10Mg/SiC resistance to impact loading and identify potential limitations associated with dynamic loading conditions.

### 2.4. Numerical Framework

#### 2.4.1. PBF-LB/M Modelling

A thermo-mechanical simulation of the PBF-LB/M process was performed using Simufact Additive software to evaluate manufacturing-induced thermal and mechanical effects on a steering knuckle component. The analysis focused on a representative geometry and assessed residual stress development, geometric distortion, and accumulated displacement resulting from the layer-by-layer manufacturing process. The CAD geometry was imported into Simufact Additive and positioned within the virtual build volume according to the selected fabrication orientation.

The manufacturing setup was configured using the Nikon SLM 280 HL (SLM Solutions GmbH, Lübeck, Germany) system available in the Simufact Additive machine library. A coupled thermo-mechanical simulation strategy was employed to evaluate the evolution of temperature and mechanical fields throughout the manufacturing sequence. The numerical discretization was refined in geometrically critical regions to improve the resolution of predicted stress and displacement gradients. The analysis considered the build sequence and subsequent removal of the component from the build plate, allowing the redistribution of residual stresses and the resulting geometric distortion after release from the substrate to be evaluated.

Post-processing focused on the distribution of residual stress, accumulated displacement, and final distorted geometry. These results provided a process-level assessment of the influence of thermal gradients, mechanical constraints, build orientation, and substrate removal on the predicted dimensional and mechanical response of the component. The Simufact Additive analysis was therefore carried out as a process-aware numerical tool to complement the experimental material characterization and structural finite element analysis by identifying manufacturing-induced effects that should be considered when evaluating the feasibility of PBF-LB/M fabrication of the optimized component.

#### 2.4.2. Structural Finite Element Model of the Steering Knuckle

In order to assess the structural applicability of the materials investigated in this study, Finite Element Analysis (FEA) was applied using a Formula SAE steering knuckle as a representative component-level case study; see Figure 4.

In a Formula SAE vehicle, the steering knuckle is subjected to combined loads arising from cornering, braking, steering, and suspension reactions. The experimentally determined mechanical properties of AlSi10Mg were incorporated into the numerical model to assess their ability to satisfy the structural requirements of the steering knuckle. The component was not considered a fully validated production design; rather, it was considered as a component-level structural benchmark to assess the feasibility of translating the experimentally characterized PBF-LB/M material systems into a realistic lightweight engineering application. Boundary conditions and applied loads were defined using representative Formula SAE suspension loading conditions [36]. The load cases considered in the analysis are summarized in Table 2 and include forces transmitted through the upper and lower suspension attachment points, steering tie-rod connection, wheel–hub interface, and braking interface.

For the braking system, calculations were based on the vehicle’s weight and the deceleration required to meet the regulatory rule for locking all four wheels, resulting in a force of 2000 N on the brackets. The calculations were based on Formula Student (FSAE) regulations.

(1) W=mg=360 kg9.81ms2≈3530 N,

*F_max_* = *Wμ_p_* ≈ 2000 N, (2)

where *W* is the weight of the vehicle (N), *m* is the mass of the vehicle (kg), *g* is the gravitational force (m/s^2^), *F*_max_ is the maximum breaking force, and *μ*_p_ is the friction coefficient (0.55).

The bearing loads at the interface with the powertrain system were determined assuming an approximate total vehicle weight of 360 kg. For the load distribution, it was considered that 80% of the weight shifts to the front wheels during braking, with an additional lateral force of 1.7 G applied during steering. This value aligns with FSAE design recommendations of 1.5 G, with an added safety factor of 0.2 G. The calculated parameters for the axial load and radial load were found to be 6000 N and 1500 N, respectively.

*F_a_* = 1.7*W* = 6000 N (3)

*F_r_* = 0.4*W* = 1500 N (4)

where *F_a_* is the axial force (N), and *F_r_* is the radial force (N).

The finite element model was solved under loading conditions. A mesh-independence study was performed to assess the numerical stability of the stress response (Table 3).

The maximum von Mises stress was monitored as a function of the minimum element size, allowing the sensitivity of the numerical solution to progressive mesh refinement to be evaluated.

Once a mesh adequacy was established, the structural response was evaluated in terms of von Mises stress, total deformation, and the location of critical stress-concentration regions. The experimentally determined effective material properties of the investigated materials were subsequently assigned to the steering knuckle model to compare their component-level structural responses under equivalent geometry, loading, and boundary conditions. The component-level analysis therefore provided a link between experimentally determined material properties and structural performance under representative Formula SAE loading conditions. The finite element results were subsequently used to identify critical load-bearing regions and establish the lightweight optimization of the component.

#### 2.4.3. Geometry Optimization

The geometry optimization was conducted using Creo Parametric, starting from the baseline steering knuckle CAD model. The objective was to minimize component mass while maintaining structural integrity under the representative Formula SAE loading conditions presented in Table 2 and Figure 5. The central bearing interface and the suspension, steering, and braking attachment regions were designated as preserved geometries to maintain dimensional compatibility and assembly functionality. Material removal was therefore restricted to the remaining design space, particularly regions with lower structural utilization.

The optimization procedure was formulated using a density-based topology optimization approach, with Penalization (SIMP) method, in which material distribution is iteratively adjusted to minimize structural compliance while satisfying prescribed mass and geometric constraints. The optimization domain excludes preserved functional interfaces, ensuring compatibility with the existing assembly.

The procedure involved identifying the principal load-transfer paths, modifying the material distribution, and evaluating the resulting geometry through finite element analysis. The same material properties, loading conditions, and boundary conditions were maintained for the baseline and optimized configurations to ensure a consistent structural comparison. The maximum von Mises stress was assessed against the experimentally determined AlSi10Mg yield strength of 224 MPa, which was adopted as the allowable stress. The safety factor was calculated as the ratio of yield strength to maximum von Mises stress, while total displacement was monitored to evaluate changes in structural stiffness.

The final optimized geometry achieved a mass reduction of 45.1%, from 1.13 to 0.62 kg. The maximum von Mises stress increased from 62 to 89 MPa, while the corresponding safety factor decreased from 3.58 to 2.52. These results demonstrate the trade-off between mass reduction and static structural performance under the investigated loading conditions.

## 3. Results and Discussion

### 3.1. Feedstock Powder Morphology and Composition

Figure 6 shows SEM micrographs and EDS spectra of feedstock powders prior to PBF-LB/M fabrication of the AlSi10Mg and AlSi10Mg/SiC samples.

The AlSi10Mg powder (Figure 6a) exhibited a predominantly spherical morphology characteristic of gas-atomized feedstock, together with a smaller fraction of elongated and irregular particles. Based on the SEM observations, particle sizes were mainly distributed within approximately 3–10 μm and 25–40 μm ranges. The corresponding EDS spectrum showed Al as the predominant element, together with Si and Mg, consistent with the expected elemental composition of the AlSi10Mg alloy. In contrast, the SiC reinforcement (Figure 6b) exhibited irregular nanoscale particles with an observed size range of approximately 30–120 nm, with a greater occurrence of particles within the 40–60 nm range, consistent with the nominal size reported by the supplier. The EDS analysis indicated C (63.85 wt.%) and Si (32.34 wt.%) as the predominant elements, together with a minor O contribution, supporting the expected composition of the SiC reinforcement. The presence of particle agglomerates was observed. This agglomeration after mixing may contribute to local heterogeneity during subsequent PBF-LB/M processing, influencing the mechanical response of the composite.

The experimentally determined relative densities were 96.07% for AlSi10Mg and 94.23% for AlSi10Mg/SiC, compared with the theoretically estimated values of 99.606% and 99.404%, respectively. The theoretical relative densities represent processing-based densification estimates rather than experimentally measured values. The lower experimental values indicate that the selected processing conditions did not eliminate porosity, particularly for the SiC-containing material. These results should therefore be considered when interpreting the subsequent mechanical response.

### 3.2. Tensile Behavior

The tensile behavior of the PBF-LB/M-fabricated materials was evaluated from stress–strain curves, as shown in Figure 7, where Figure 7a corresponds to the AlSi10Mg alloy and Figure 7b to AlSi10Mg/SiC.

The experimental curves reveal marked differences in the tensile response of the two materials (Table 4). The AlSi10Mg alloy exhibited an ultimate tensile strength of 362.39 ± 4.61 MPa and a Young’s modulus of 60.68 ± 1.94 GPa. The low elastic modulus value is partially associated with processing-induced porosity and microstructural heterogeneity. In comparison, the AlSi10Mg/SiC composite exhibited an ultimate tensile strength of 219.79 ± 7.86 MPa and a Young’s modulus of 52.47 ± 5.18 GPa. Thus, under the processing conditions investigated, the incorporation of SiC did not result in an improvement in tensile performance.

The reduced strength and ductility of the composite are consistent with the microstructural heterogeneities observed in the characterization, including the tendency of the reinforcement to form agglomerates. Such heterogeneities, together with process-induced defects and possible matrix–reinforcement interfacial discontinuities, can act as local stress concentrators and promote premature damage initiation, thereby limiting the effective contribution of the reinforcement. It is important to acknowledge that the AlSi10Mg and AlSi10Mg/SiC specimens were fabricated using different PBF-LB/M processing parameters, corresponding to volumetric energy densities of approximately 57 and 103 J/mm^3^, respectively. Consequently, the observed differences in tensile performance cannot be attributed exclusively to the incorporation of SiC reinforcement, as variations in processing conditions may also influence microstructural evolution and mechanical properties.

### 3.3. Comparative High-Cycle Fatigue Behavior

High-cycle fatigue tests were conducted under fully reversed loading conditions (R = −1) at a frequency of 50 Hz, with 10^7^ cycles established as the run-out criterion. The fatigue tests were designed as a comparative assessment at selected normalized stress levels rather than as a complete S–N characterization. The applied stress amplitudes were defined as fractions of the experimentally determined yield strength of each material. For the AlSi10Mg alloy (*σ_y_* = 224 MPa), a stress amplitude of 96 MPa, corresponding to approximately 43% of *σ_y_*, was evaluated.

For AlSi10Mg/SiC, stress amplitudes of 84 and 96 MPa, corresponding to approximately 43% and 50% of its experimentally determined yield strength, respectively, were investigated. The AlSi10Mg alloy reached the run-out criterion of 10^7^ cycles at 96 MPa without failure, whereas the AlSi10Mg/SiC composite failed at approximately 8 × 10^6^ cycles under the same stress amplitude (Figure 8). It should be noted that the limited number of stress levels investigated does not allow the construction of statistically representative S–N curves or the determination of fatigue endurance limits.

### 3.4. Impact Behavior and Fractography

The impact behavior of the PBF-LB/M-fabricated AlSi10Mg/SiC was evaluated by Charpy impact testing, and the absorbed energy values are summarized in Table 5.

The impact specimens exhibited an average absorbed energy of 3.88 ± 0.64 J, with relatively limited variation among the measurements. The comparatively low absorbed energy indicates a limited capacity for energy dissipation under impact loading and is consistent with a predominantly brittle fracture response. In PBF-LB/M-fabricated materials, impact behavior can be influenced by process-induced microstructural heterogeneities, including porosity, melt-pool-related features, and local variations generated by rapid solidification. In order to further examine the associated fracture mechanisms, representative fracture surfaces were analyzed (Figure 9).

The fracture surfaces exhibited predominantly brittle characteristics, with relatively flat regions and limited evidence of plastic deformation. Local features consistent with particle pull-out and matrix–reinforcement interfacial separation were also observed. These features suggest that reinforcement distribution and local interfacial integrity may contribute to the fracture response by providing preferential sites for damage initiation and propagation. The impact response of the composite under the investigated PBF-LB/M processing conditions identifies limited impact energy absorption as an important consideration for applications involving sudden or impact loading.

### 3.5. PBF-LB/M Process Simulation

In order to complement the experimental characterization, a thermo-mechanical process simulation was performed using Simufact Additive to evaluate manufacturing-induced stress and distortion during PBF-LB/M fabrication of the steering knuckle; see Figure 10. The simulation was performed using AlSi10Mg as the material model. Accordingly, the simulation should be interpreted as a process-level assessment of the AlSi10Mg steering knuckle.

The first stage of the analysis consisted of generating the computational discretization of the steering knuckle geometry, as shown in Figure 10a. A voxel-based discretization with a voxel size of 2.72 mm and two levels of coarsening was employed to provide a compromise between computational cost and geometric representation. Particular attention was given to the representation of support interfaces, mounting features, and relatively thin regions of the geometry because these features can influence the predicted thermo-mechanical response during the simulated build sequence. Following completion of the build simulation, the response after support and base-plate release was evaluated in terms of total displacement and equivalent stress, as shown in Figure 10b,c.

The predicted displacement field exhibited a non-uniform distribution across the component, with the largest deformation occurring in the upper regions and other geometrically less constrained features. A maximum total displacement of approximately 10.62 mm was predicted after release. The magnitude and spatial distribution of the displacement indicate a pronounced sensitivity of the simulated geometry to the release of manufacturing constraints. These results suggest that build orientation, support configuration, and constraint conditions are important considerations for controlling the dimensional stability of the component during PBF-LB/M fabrication.

The equivalent stress distribution after release is presented in Figure 10c. Regions of elevated stress were primarily observed around geometric discontinuities, including hole boundaries, mounting features, and transitions between the main body and protruding sections of the steering knuckle. A maximum equivalent stress of approximately 370 MPa was predicted. The predicted displacement and equivalent stress fields demonstrate that the PBF-LB/M manufacturing sequence can introduce substantial geometric distortion and non-uniform residual stresses in the steering knuckle geometry. In particular, the large, predicted displacement after release indicates that the initially selected build configuration would require further optimization of build orientation, support strategy, and/or process conditions before physical fabrication of the component. These results therefore provide a process-level assessment of manufacturing feasibility rather than a direct prediction of the final structural performance of the steering knuckle.

The process simulation complements the experimental material characterization and subsequent structural analysis by identifying manufacturing-induced effects that are not captured when component performance is evaluated using nominal or experimentally determined material properties alone. The results emphasize the importance of considering manufacturing history, component geometry, support conditions, and constraint release when assessing the feasibility of PBF-LB/M-fabricated lightweight components.

The process simulation provides a preliminary assessment of manufacturing-induced stresses and distortions. Its accuracy is limited by constitutive modeling assumptions and coarse voxel discretization. As neither numerical convergence nor experimental validation of the predicted residual stresses and distortions was established, the results should be interpreted as qualitative indicators of manufacturing feasibility rather than validated quantitative predictions. The subsequent FEA and geometry optimization were therefore performed on the nominal CAD geometry to independently assess structural performance and lightweight potential under representative service loads.

### 3.6. Baseline Steering Knuckle Structural Response

The structural response of the Formula SAE steering knuckle was evaluated in ANSYS (2026 R1) under the representative loading conditions defined in Table 2. The applied load cases included forces transmitted through the suspension, steering, braking, and bearing interfaces, while the corresponding constraints were imposed at the functional connection regions according to the boundary conditions described in Section 2. Figure 11 presents the finite element results in terms of (a) total deformation, (b) von Mises stress, and (c) safety factor under the combined static loading conditions.

The total deformation field (Figure 11a) shows a maximum value of approximately 0.11 mm, located primarily at the upper mounting region. The deformation progressively decreases toward the more constrained regions of the component. The relatively small displacement compared with the overall dimensions of the steering knuckle indicates that the baseline geometry maintains adequate global stiffness under the evaluated static loading conditions. The von Mises stress distribution (Figure 11b) exhibits a maximum value of approximately 62 MPa. The highest stress levels are concentrated around geometrical discontinuities and load-transfer regions, particularly near the central bearing interface, fillets, and transitions between the main body and the mounting arms. These locations are consistent with the expected stress amplification associated with changes in cross-section and multiaxial load transfer. In contrast, a substantial portion of the component remains at considerably lower stress levels, indicating that material is not uniformly utilized throughout the baseline geometry.

The safety factor distribution (Figure 11c) shows a minimum value of approximately 3.58 in the most highly stressed regions, while substantially higher values are observed over most of the component. The simultaneous presence of localized stress concentrations and extensive regions with comparatively low stress suggests that the baseline design contains opportunities for material redistribution and mass reduction while preserving the principal load-bearing paths. The finite element results indicate that the baseline steering knuckle satisfies the evaluated static structural criteria, with a maximum deformation of approximately 0.11 mm, a maximum von Mises stress of approximately 62 MPa, and a minimum safety factor of approximately 3.58. These results establish the structural reference state for the subsequent geometry optimization.

### 3.7. Geometry Optimization and Lightweight Design

To improve the structural efficiency of the steering knuckle, a geometry optimization strategy based on generative design principles was implemented using Creo Parametric. The optimization framework generated and evaluated alternative geometrical configurations according to the prescribed material properties, functional constraints, preserved interfaces, and representative loading conditions. The objective was to minimize component mass while maintaining the principal load-transfer paths and satisfying the established structural requirements. The optimization resulted in a mass reduction of approximately 45%, decreasing the component mass from approximately 1.13 kg for the initial geometry to 0.62 kg for the optimized configuration. Material was preferentially removed from structurally underutilized regions while the bearing interface and suspension, steering, and braking attachment regions were preserved. As a result, the optimized geometry exhibited a more material-efficient configuration in which the remaining material was concentrated along the principal load-bearing paths, as illustrated in Figure 12.

Following geometry optimization, the structural response of the optimized steering knuckle was evaluated by applying finite element analysis under the same representative loading conditions considered for the baseline configuration. Figure 13 presents the von Mises stress distribution and the total displacement field obtained for the optimized AlSi10Mg steering knuckle.

As shown in Figure 13a, the optimized geometry exhibited a non-uniform stress distribution, with the highest stresses concentrated around geometrical discontinuities, mounting interfaces, and transitions between the main body and the load-transfer arms. The maximum von Mises stress was approximately 89 MPa. Compared with the baseline configuration, for which a maximum stress of approximately 62 MPa was predicted, the optimized design exhibited a 42.2% increase in localized stress due to substantial material removal and redistribution. Nevertheless, the maximum stress remained below the experimentally determined yield strength of AlSi10Mg (224 MPa), resulting in a minimum safety factor of 2.52, calculated as the ratio of yield strength to maximum von Mises stress.

The corresponding displacement field is shown in Figure 13b. The maximum total displacement was approximately 0.32 mm and occurred primarily at the less constrained extremities and mounting features. Despite the substantial reduction in component mass, the predicted displacement remained relatively small compared with the overall dimensions of the steering knuckle, indicating that the optimized geometry retained adequate global stiffness under the investigated static loading conditions. The comparison between the baseline and optimized configurations reveals the expected trade-off between mass reduction and structural response (Table 6). The 45.1% reduction in mass, from 1.13 to 0.62 kg, increased the maximum von Mises stress from 62 to 89 MPa. The minimum safety factor consequently decreased from 3.58 to 2.52, using the same allowable stress for both configurations (Figure 13c). These results indicate that the optimization redistributed material toward the principal load-transfer paths while removing material from less structurally utilized regions.

Despite a 191% increase in displacement, the optimized design exhibited a maximum deformation of only 0.32 mm, suggesting that sufficient structural stiffness was retained under the evaluated loading conditions while achieving a 45.1% mass reduction. However, these results should be interpreted as a numerical demonstration of lightweight design feasibility rather than as experimental validation of the final component.

A preliminary fatigue screening was performed in ANSYS Mechanical using the experimentally observed run-out condition at 10^7^ cycles as the available fatigue reference (Figure 14).

The resulting minimum fatigue safety factor of approximately 1.08 was used only to identify potentially fatigue-critical regions of the optimized geometry. Because a single run-out point does not constitute an S–N curve or establish an endurance limit, this value should not be interpreted as a validated fatigue safety factor or fatigue-life prediction. Additional fatigue tests at multiple stress amplitudes and with replicate specimens are required for quantitative component-level fatigue assessment.

### 3.8. Integrated Process–Material–Component Discussion

The results demonstrate the potential of generative design for improving the structural efficiency of lightweight automotive components. The optimized steering knuckle achieved an approximately 45% mass reduction while maintaining a minimum safety factor of 2.52 and limited deformation under the evaluated static loading conditions. Material removal was concentrated in less structurally utilized regions while the principal load paths and functional interfaces were preserved.

The resulting reduction in mass is particularly relevant for Formula SAE applications, while the geometric freedom provided by PBF-LB/M enables the fabrication of complex optimized geometries that would be difficult to achieve using conventional manufacturing. The process simulation predicted substantial residual stresses and distortion, indicating that build orientation, support strategy, and process conditions must also be considered before physical fabrication. Thus, structural and manufacturing optimization should be treated as complementary stages of the design process.

The combined experimental and numerical results highlight the importance of considering the interaction between PBF-LB/M processing, material behavior, and component design. At the process scale, Simufact Additive predicted non-uniform residual stresses and substantial geometric distortion after support and base-plate release, demonstrating the sensitivity of the steering knuckle geometry to manufacturing conditions. At the material scale, the expected strengthening effect of SiC was not fully realized. Compared with the AlSi10Mg base alloy, the composite exhibited lower tensile strength and ductility, while the comparative fatigue assessment showed no clear improvement in high-cycle fatigue performance. Charpy testing of the composite resulted in an average absorbed energy of 3.88 ± 0.64 J, and SEM fractography revealed predominantly brittle features together with particle pull-out and local matrix–reinforcement interfacial separation.

At the component scale, finite element analysis showed that the baseline steering knuckle satisfied the evaluated static structural criteria. Although the optimized geometry exhibited higher localized stresses, its predicted deformation remained limited, demonstrating a more efficient use of material. The results show that the potential of PBF-LB/M for lightweight structural applications must consider material behavior, manufacturing constraints, and component-level performance.

## 4. Conclusions

This study presented an integrated experimental–numerical framework for evaluating PBF-LB/M-fabricated AlSi10Mg and AlSi10Mg/SiC materials and their potential application to the lightweight design of a Formula SAE steering knuckle. The main conclusions are as follows:

1. The incorporation of SiC particles did not translate into an overall improvement in the mechanical performance of AlSi10Mg under the processing conditions investigated. For SiC reinforcement, microstructural characterization revealed particle agglomeration and local heterogeneities, while fractographic observations identified features consistent with particle pull-out and interfacial separation.
2. The PBF-LB/M process simulation identified significant manufacturing-induced residual stresses and geometric distortion in the steering knuckle geometry. After support and base-plate release, a maximum displacement of approximately 10.62 mm and localized equivalent stresses of approximately 370 MPa were predicted. These results indicate that building orientation, support configuration, and constraint release are important factors governing the manufacturing feasibility and dimensional stability of the component. Further optimization of the manufacturing strategy would therefore be required before physical fabrication.
3. The experimental characterization demonstrated a strong dependence of mechanical response on the investigated material and PBF-LB/M processing conditions. AlSi10Mg/SiC exhibited lower tensile strength and ductility than unreinforced AlSi10Mg; however, because different processing parameters were used for the two materials, this reduction cannot be attributed exclusively to SiC reinforcement. The limited high-cycle fatigue tests provided a comparative assessment at selected stress amplitudes rather than a complete S–N characterization. Charpy impact testing of the AlSi10Mg/SiC composite resulted in an average absorbed energy of 3.88 ± 0.64 J and predominantly brittle fracture features.
4. Component-level numerical analyses were conducted exclusively for monolithic AlSi10Mg. Geometry optimization reduced the steering-knuckle mass from approximately 1.13 to 0.62 kg, corresponding to a 45.1% reduction. Under the static loading conditions investigated, the optimized configuration exhibited a maximum von Mises stress of approximately 89 MPa and a minimum static safety factor of 2.52, compared with 62 MPa and 3.58 for the baseline design. A preliminary fatigue screening using the limited experimental fatigue data identified potentially critical regions; however, it should not be interpreted as a validated fatigue-life prediction because a statistically representative S–N curve was not available.
5. The results demonstrate the importance of integrating experimental material characterization, manufacturing-process simulation, structural analysis, and geometry optimization in the development of lightweight PBF-LB/M components. The Simufact analysis identified manufacturing-related residual-stress and distortion risks associated with the initial build configuration, while the structural analysis independently assessed the nominal geometry under representative service loads. The optimized steering knuckle therefore represents a lightweight design concept rather than a fully validated component, requiring further PBF-LB/M process optimization, comprehensive fatigue characterization, physical fabrication, and component-level experimental validation.

## Figures and Tables

**Figure 1:**
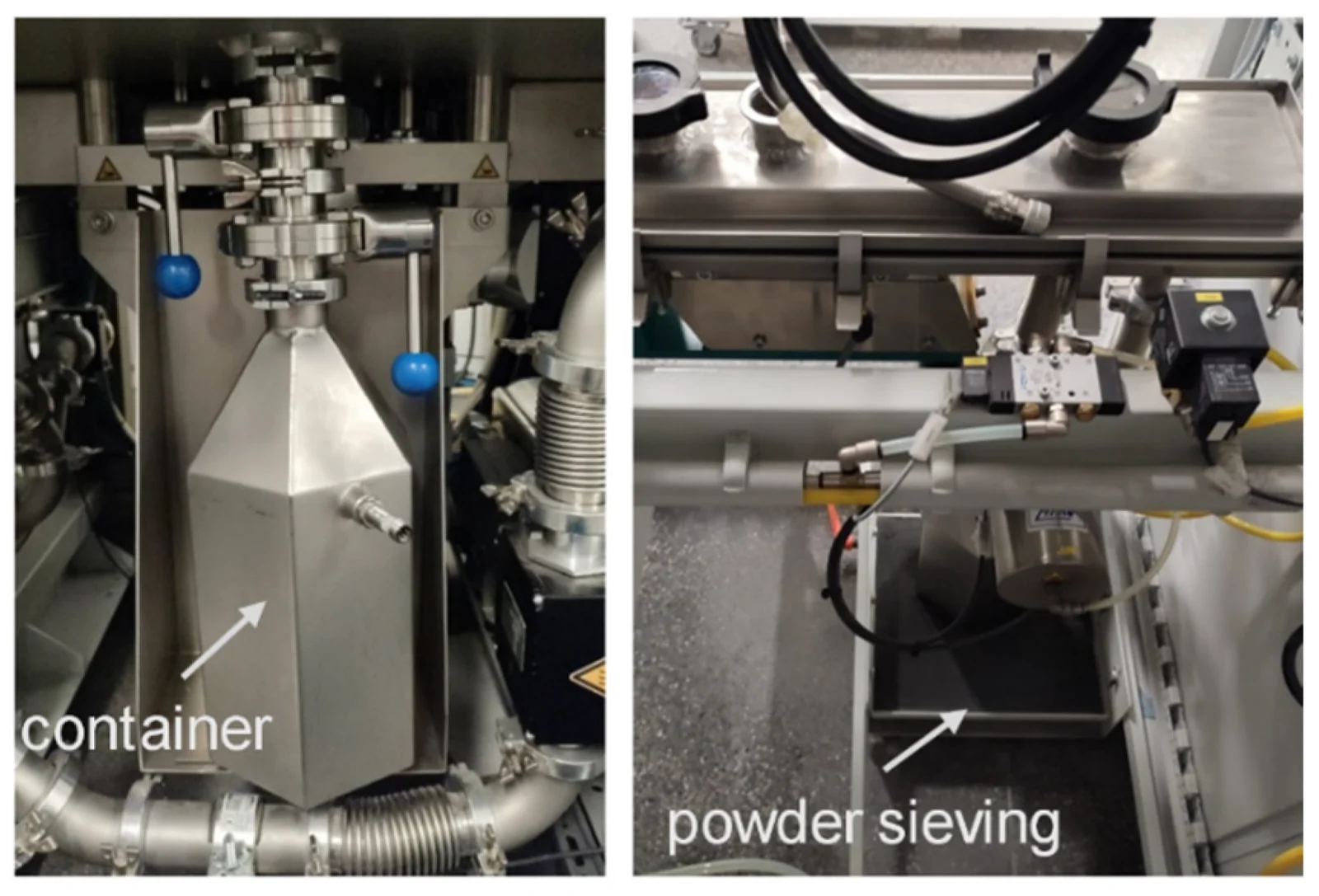
Powder preparation and sieving process with the PSM 100 system.

**Figure 2:**
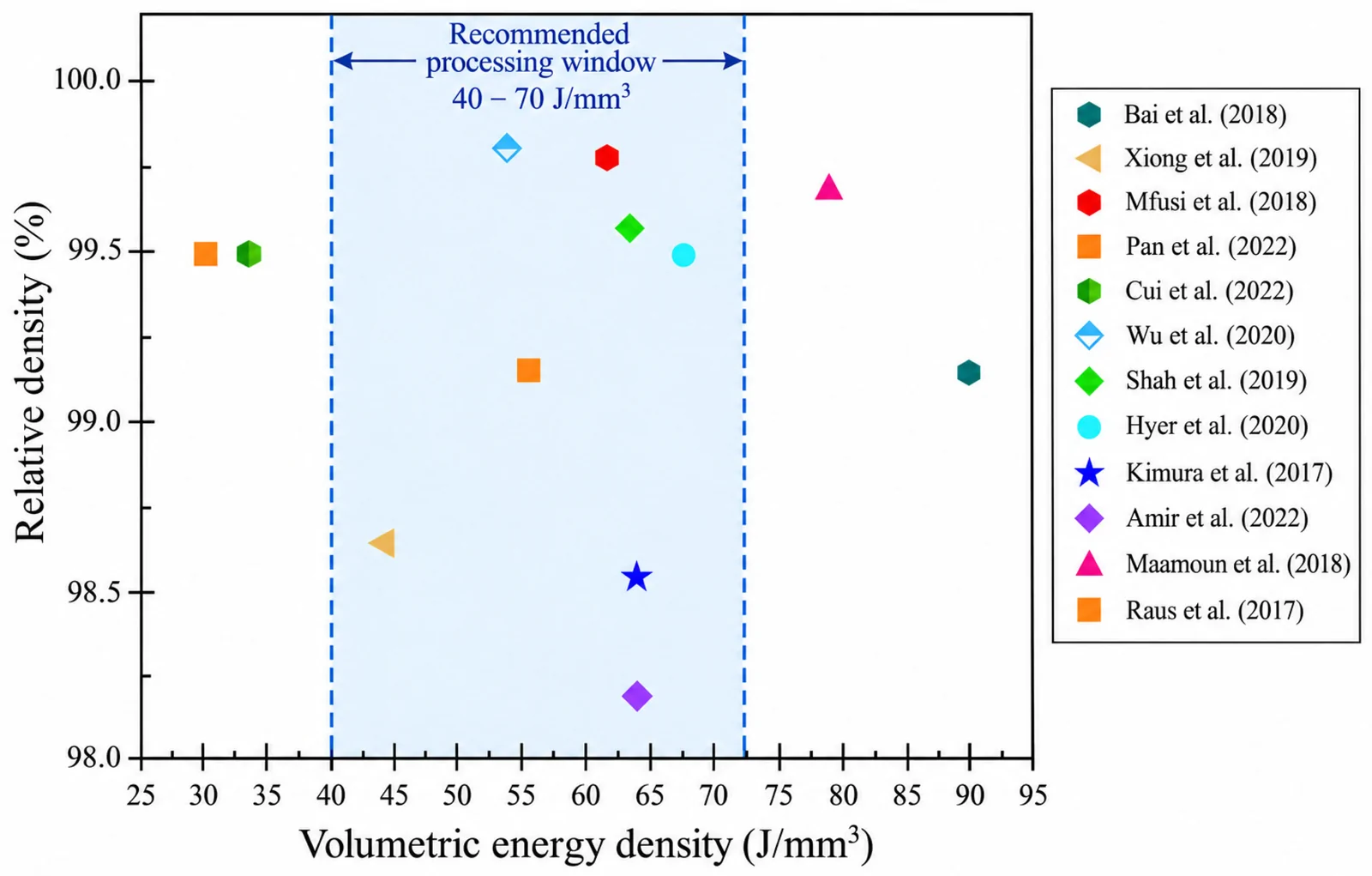
Comparison of relative density as a function of volumetric energy density (VED) for laser powder bed fusion of aluminum alloys, based on previously published experimental studies: Bai et al. [17], Xiong et al. [18], Mfusi et al. [19], Pan et al. [20], Cui et al. [21], Wu et al. [22], Shah and Dey [23], Hyer et al. [24], Kimura et al. [25], Amir et al. [26], Maamoun et al. [27], and Raus et al. [28]. The shaded region indicates the recommended processing window of 40–70 J/mm^3^.

**Figure 3:**
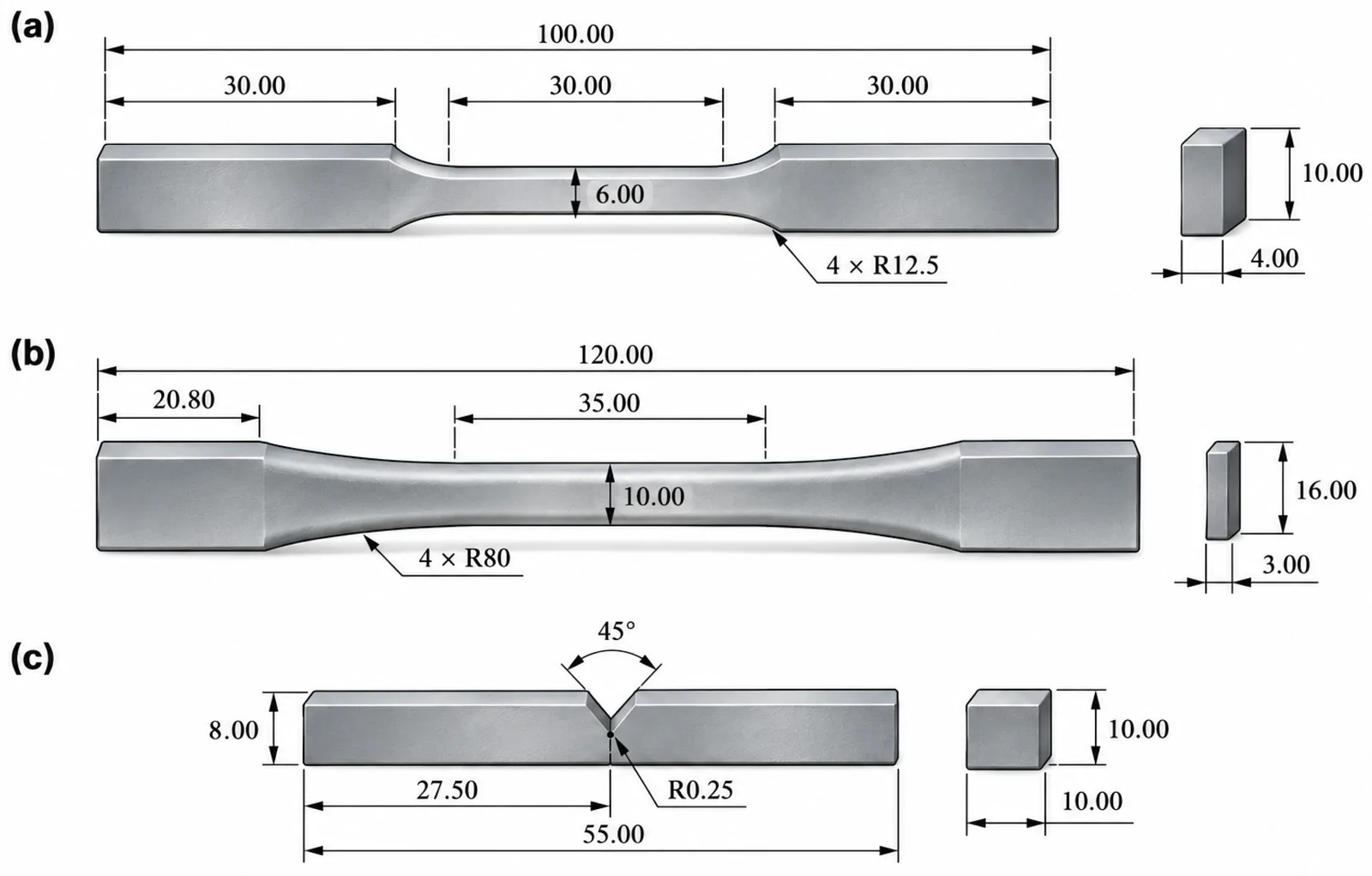
Specimen geometries and dimensions (mm) used for mechanical characterization: (**a**) tensile testing, (**b**) axial fatigue testing, and (**c**) Charpy impact testing.

**Figure 4:**
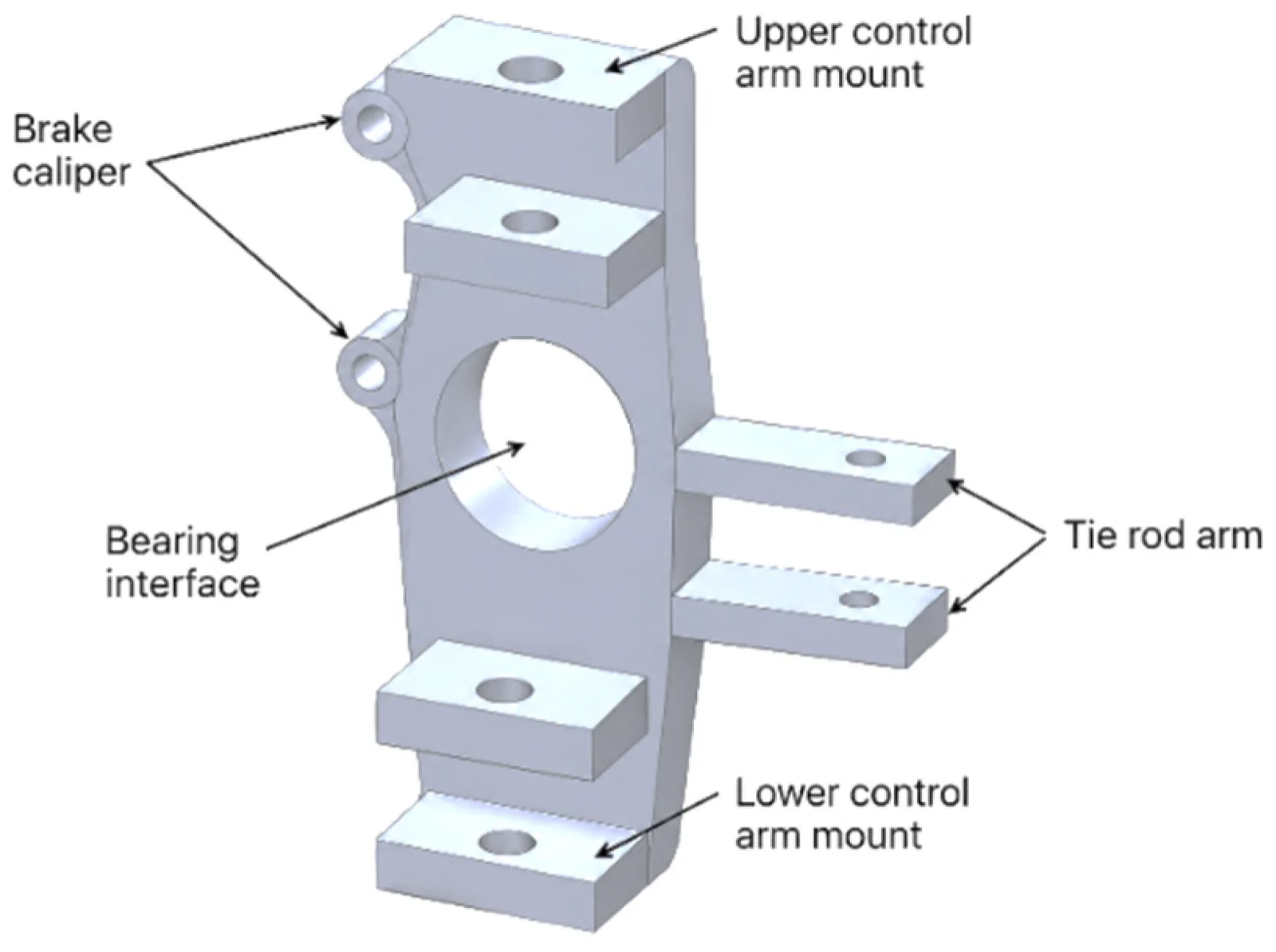
Geometry of the Formula SAE steering knuckle showing the main functional interfaces and load-transfer regions.

**Figure 5:**
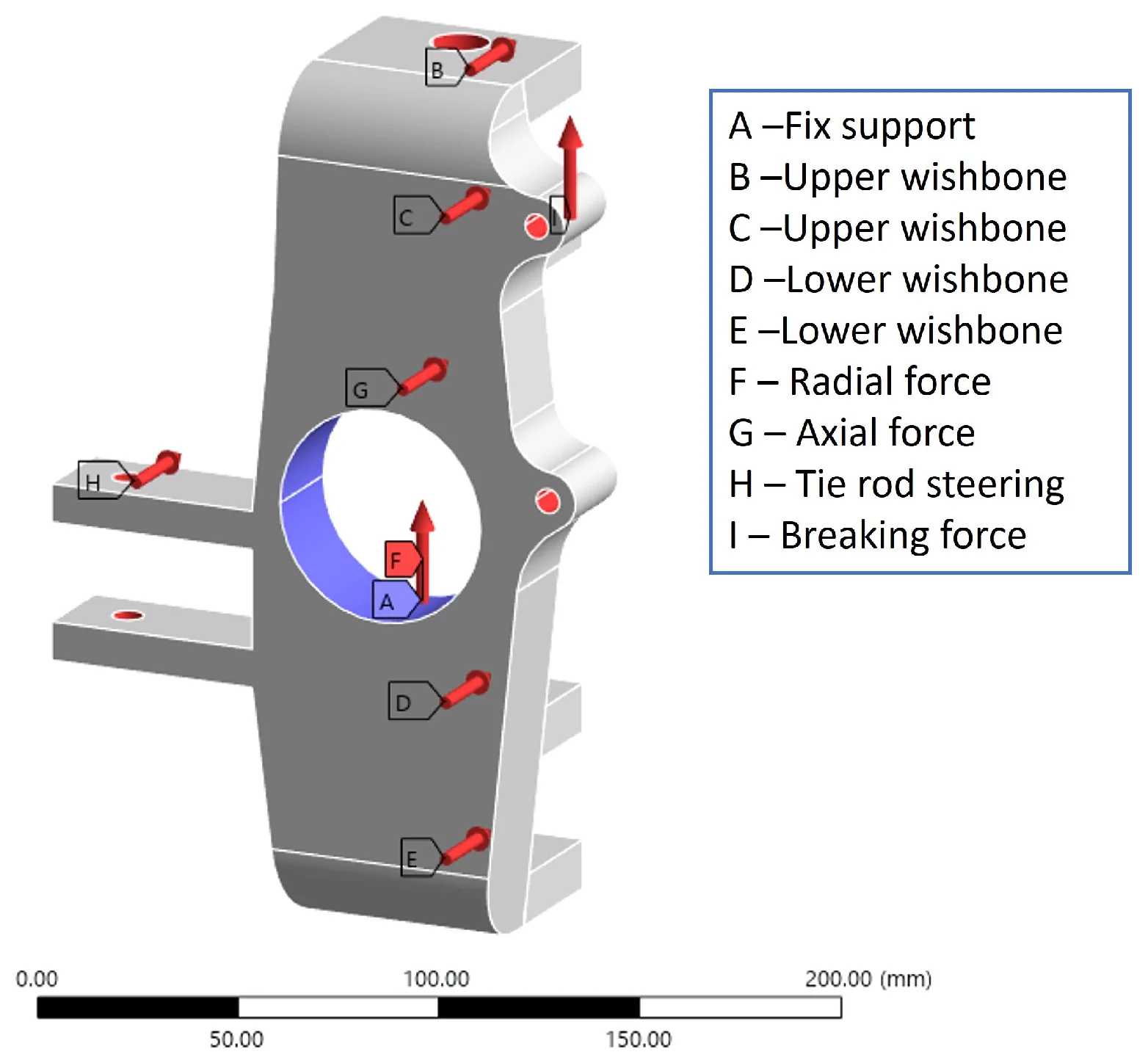
Boundary conditions and loading configuration applied to the steering knuckle during the structural finite element and geometry optimization analyses.

**Figure 6:**
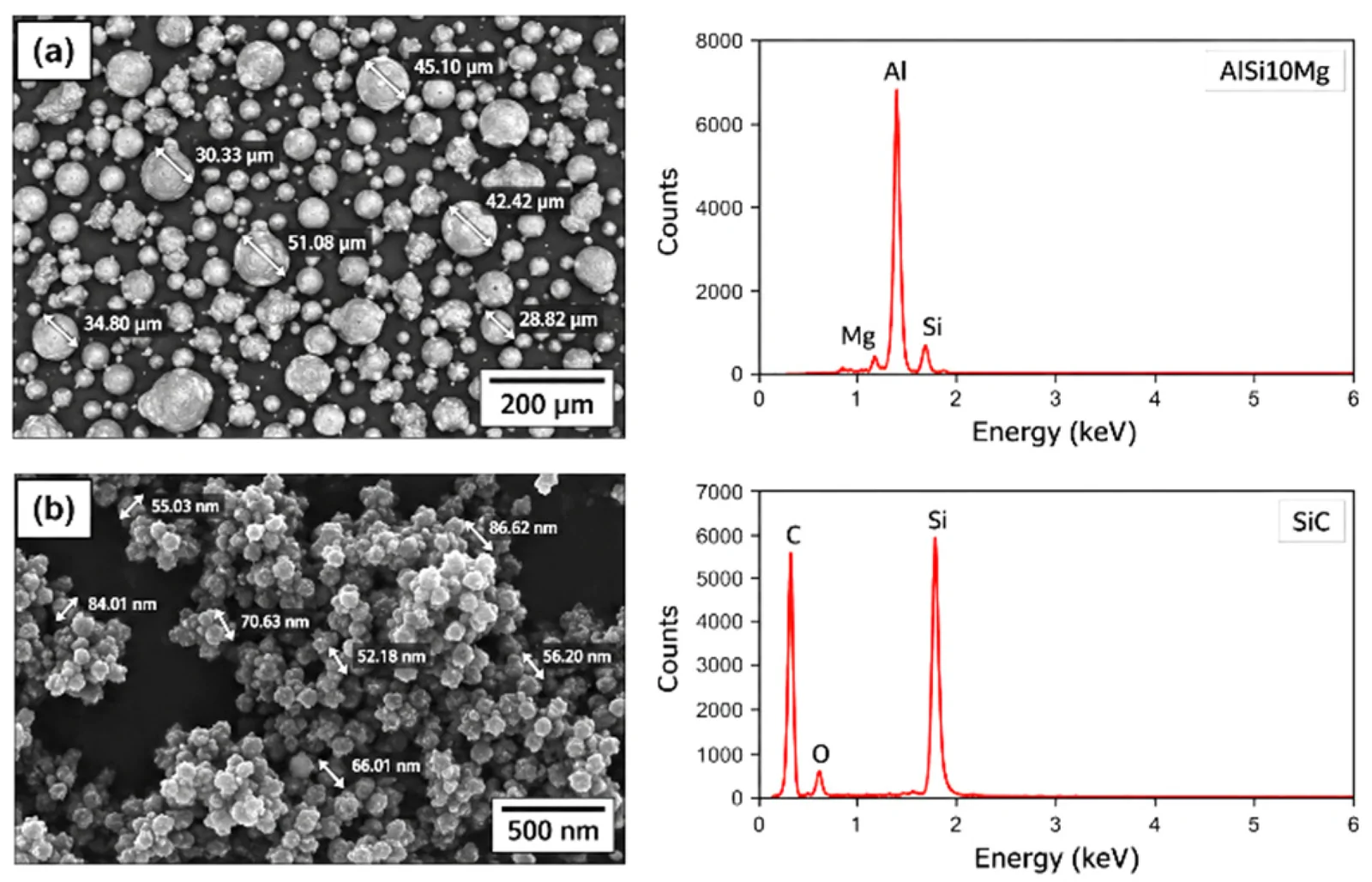
SEM micrographs and EDS spectra of the feedstock powders: (**a**) AlSi10Mg powder exhibiting predominantly spherical particles with a broad particle-size distribution; and (**b**) SiC reinforcement showing irregular nanoscale morphology and particle agglomeration.

**Figure 7:**
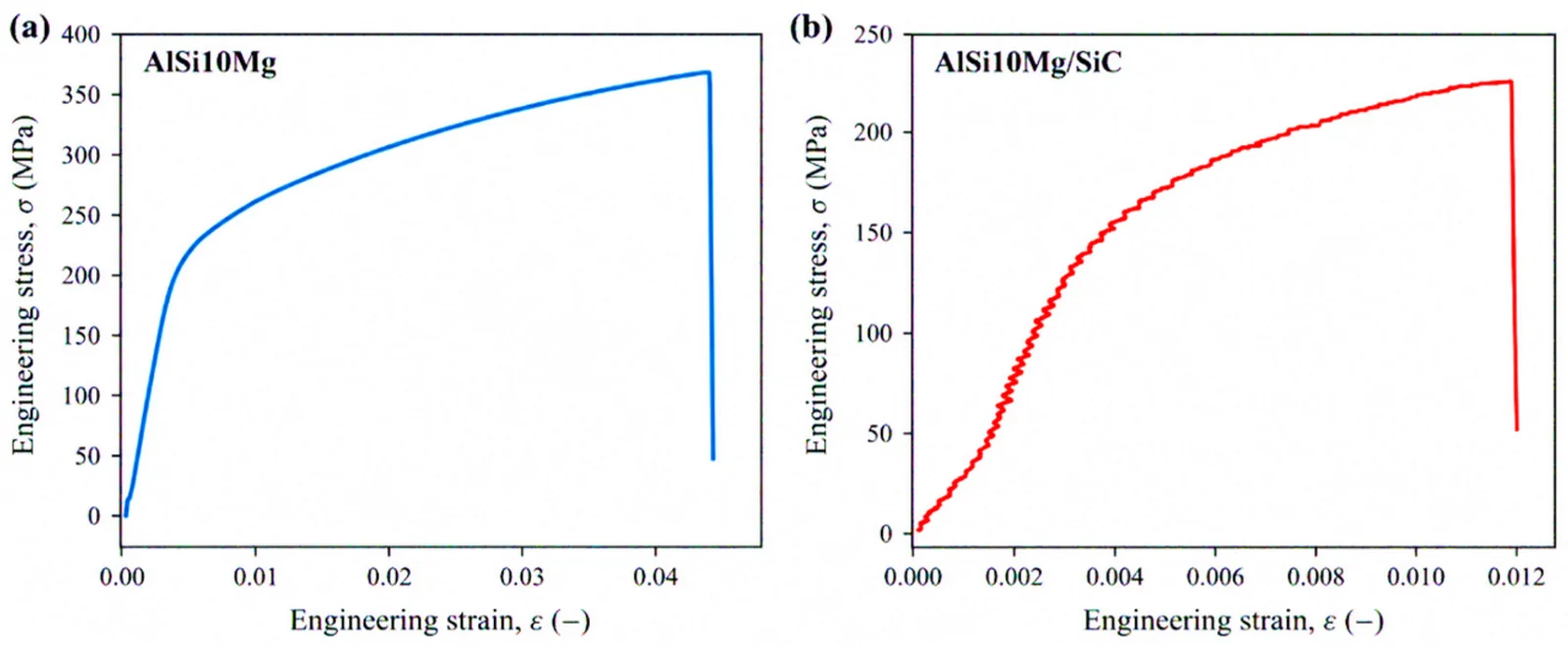
Stress–strain curves obtained from tensile testing of the PBF-LB/M-fabricated materials: (**a**) AlSi10Mg base alloy and (**b**) AlSi10Mg/SiC.

**Figure 8:**
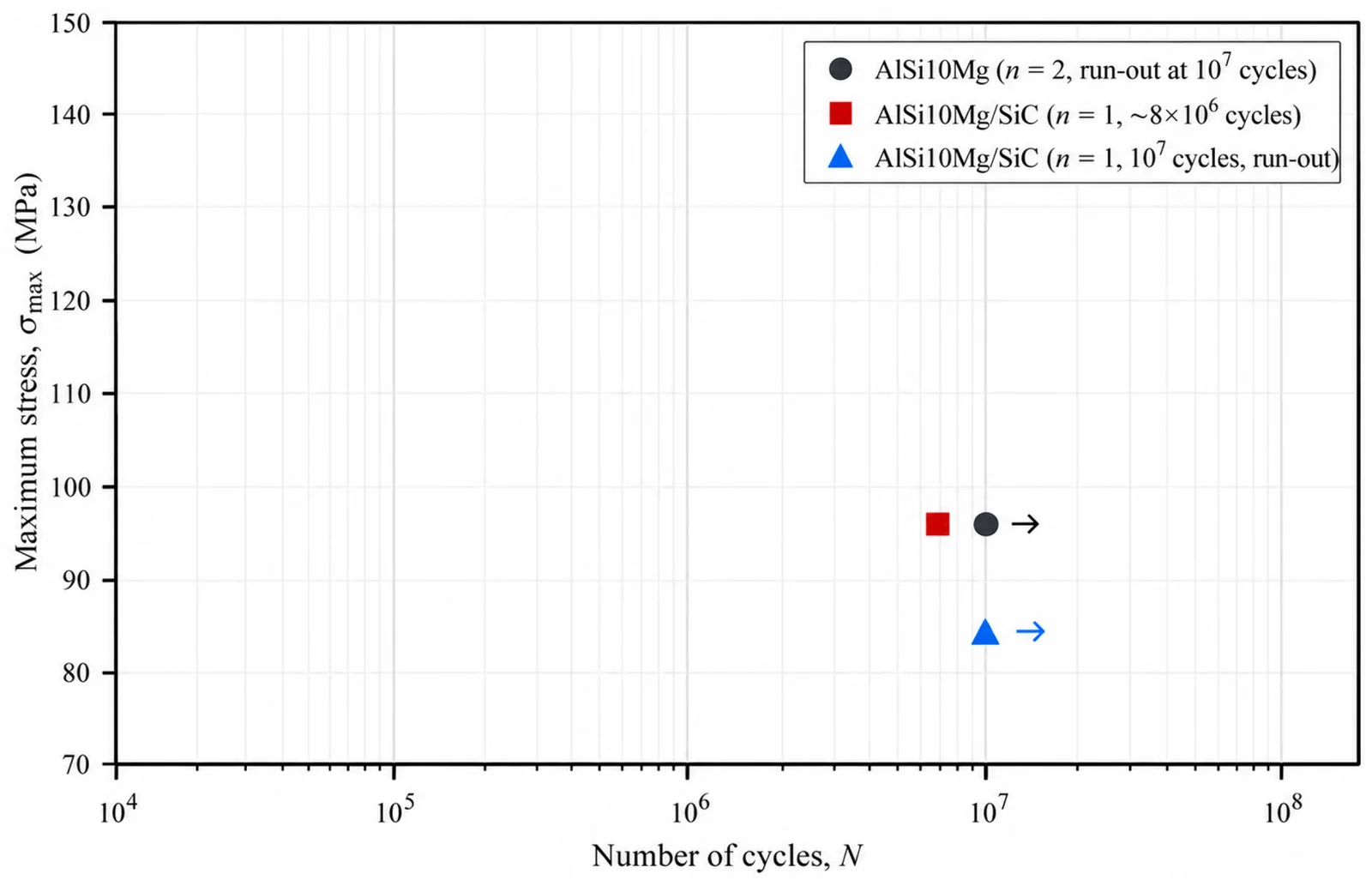
Comparative high-cycle fatigue response of AlSi10Mg and AlSi10Mg/SiC (1.4 vol.% SiC) under fully reversed loading (R = −1).

**Figure 9:**
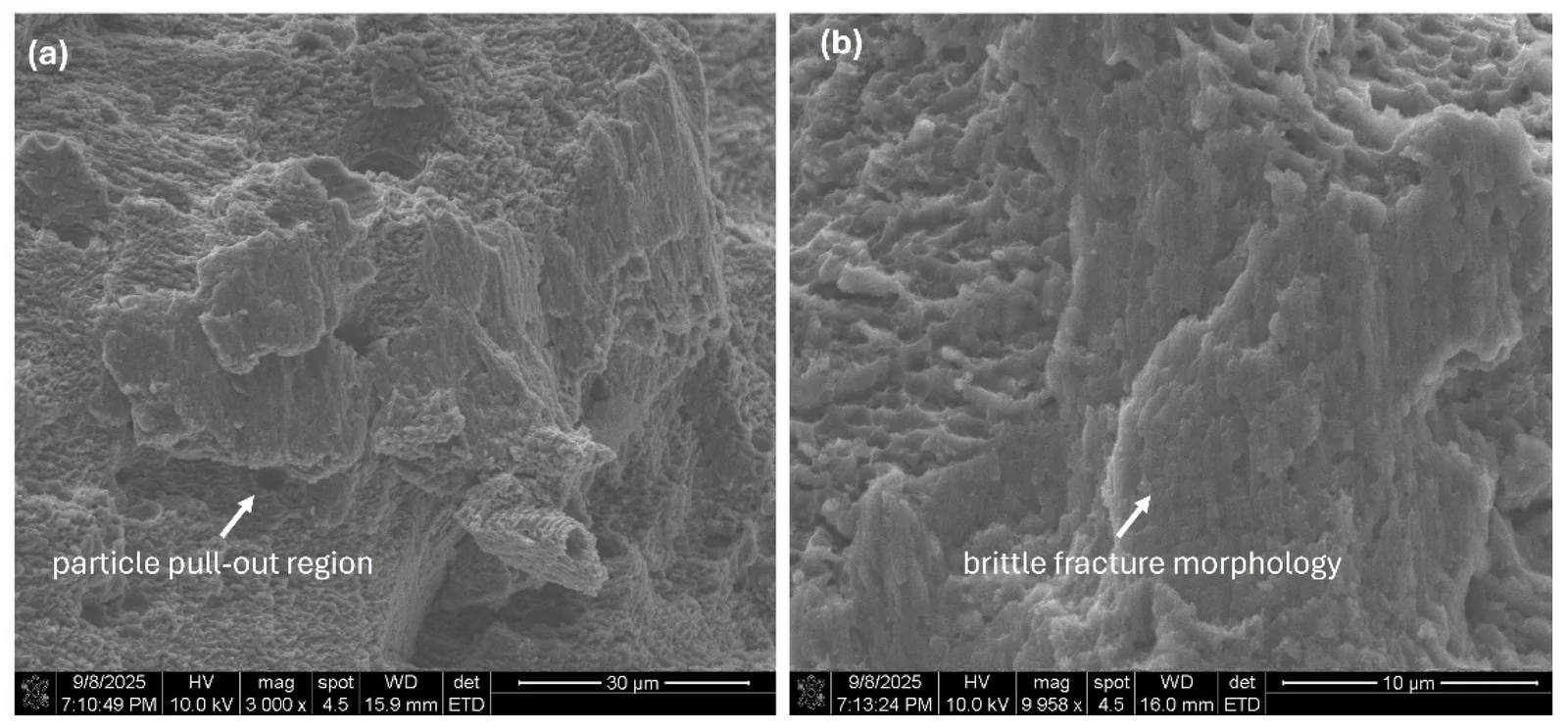
SEM fractographs of AlSi10Mg/SiC specimens after Charpy impact testing: (**a**) region exhibiting particle pull-out and local matrix–reinforcement interfacial separation; and (**b**) predominantly brittle fracture morphology characterized by relatively flat fracture features and limited evidence of plastic deformation.

**Figure 10:**
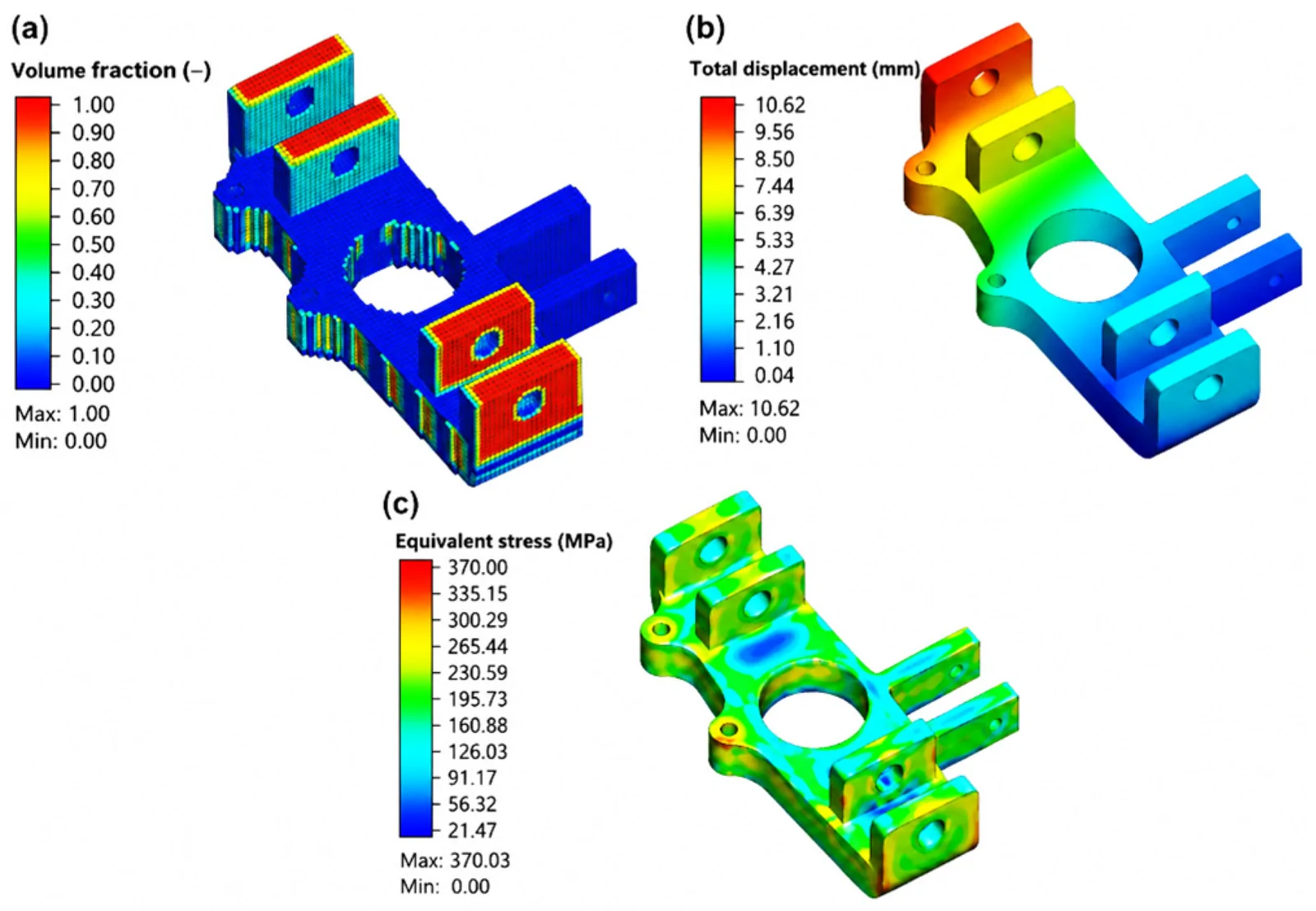
Simufact Additive process-simulation results for the PBF-LB/M fabrication of the steering knuckle: (**a**) voxel-based computational discretization, (**b**) predicted total displacement after support and base-plate release, and (**c**) equivalent stress distribution after release.

**Figure 11:**
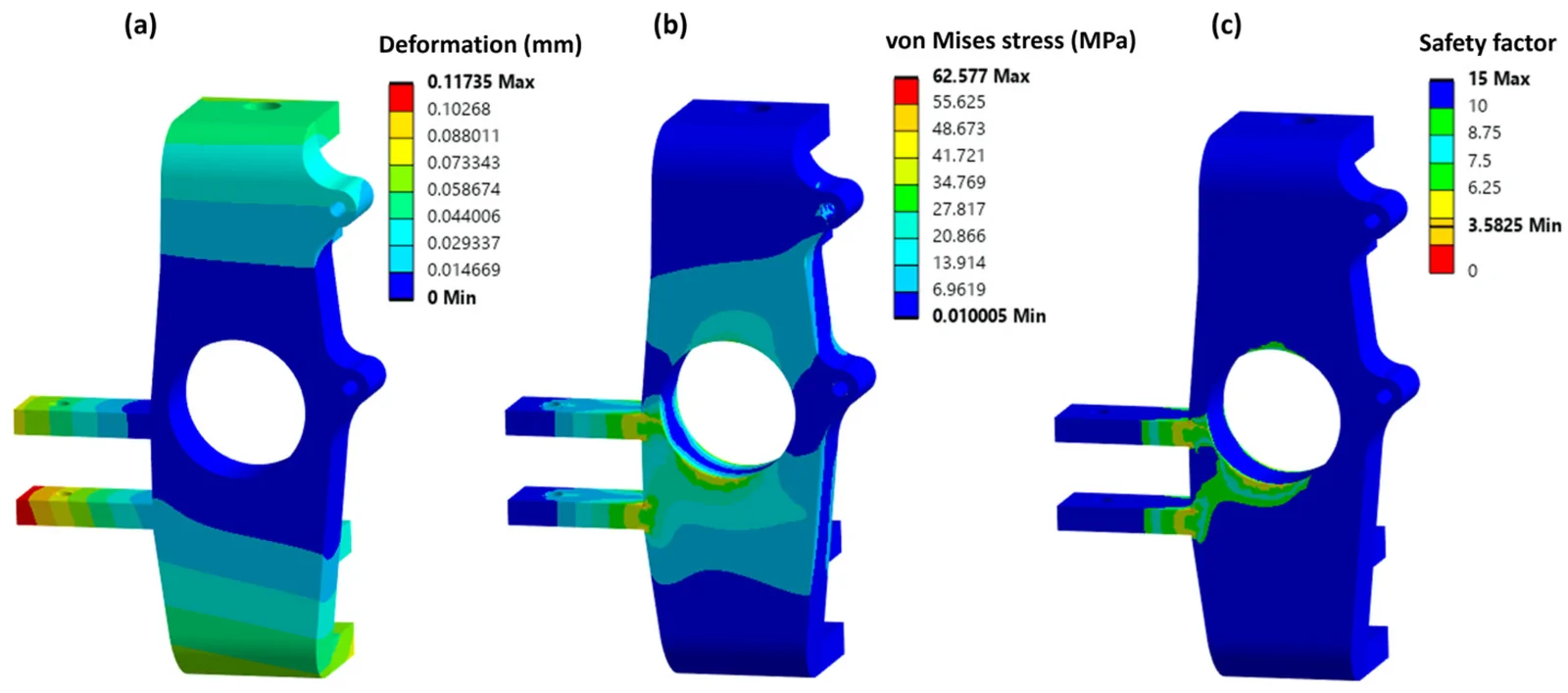
Finite element results for the Formula SAE steering knuckle under the evaluated combined static loading conditions: (**a**) total deformation (mm), (**b**) von Mises stress (MPa), and (**c**) safety factor.

**Figure 12:**
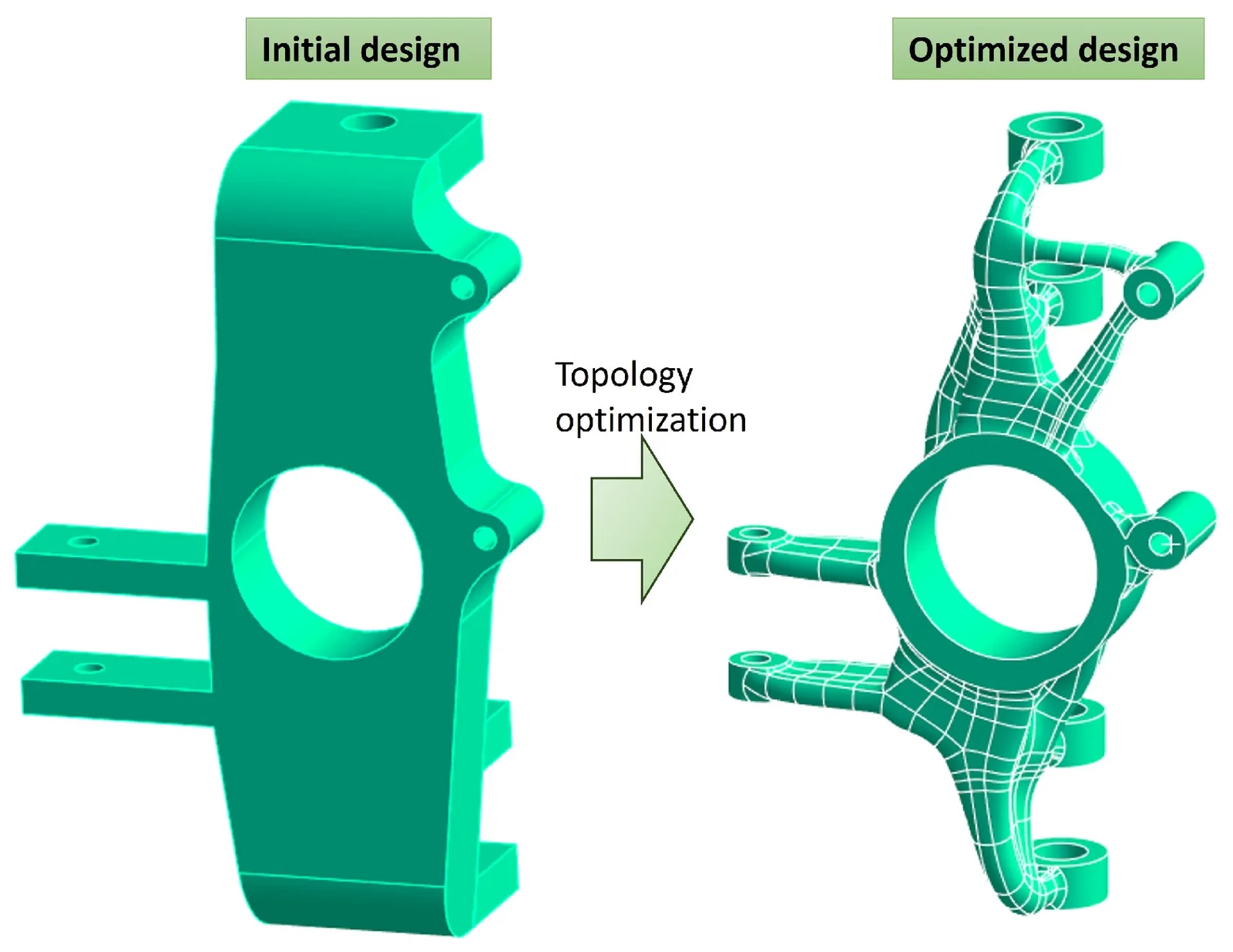
Geometry optimization of the Formula SAE steering knuckle in Creo Parametric Generative Design: initial design (**left**) and optimized geometry (**right**). Functional interfaces and principal load-transfer regions were preserved while material was redistributed to reduce component mass.

**Figure 13:**
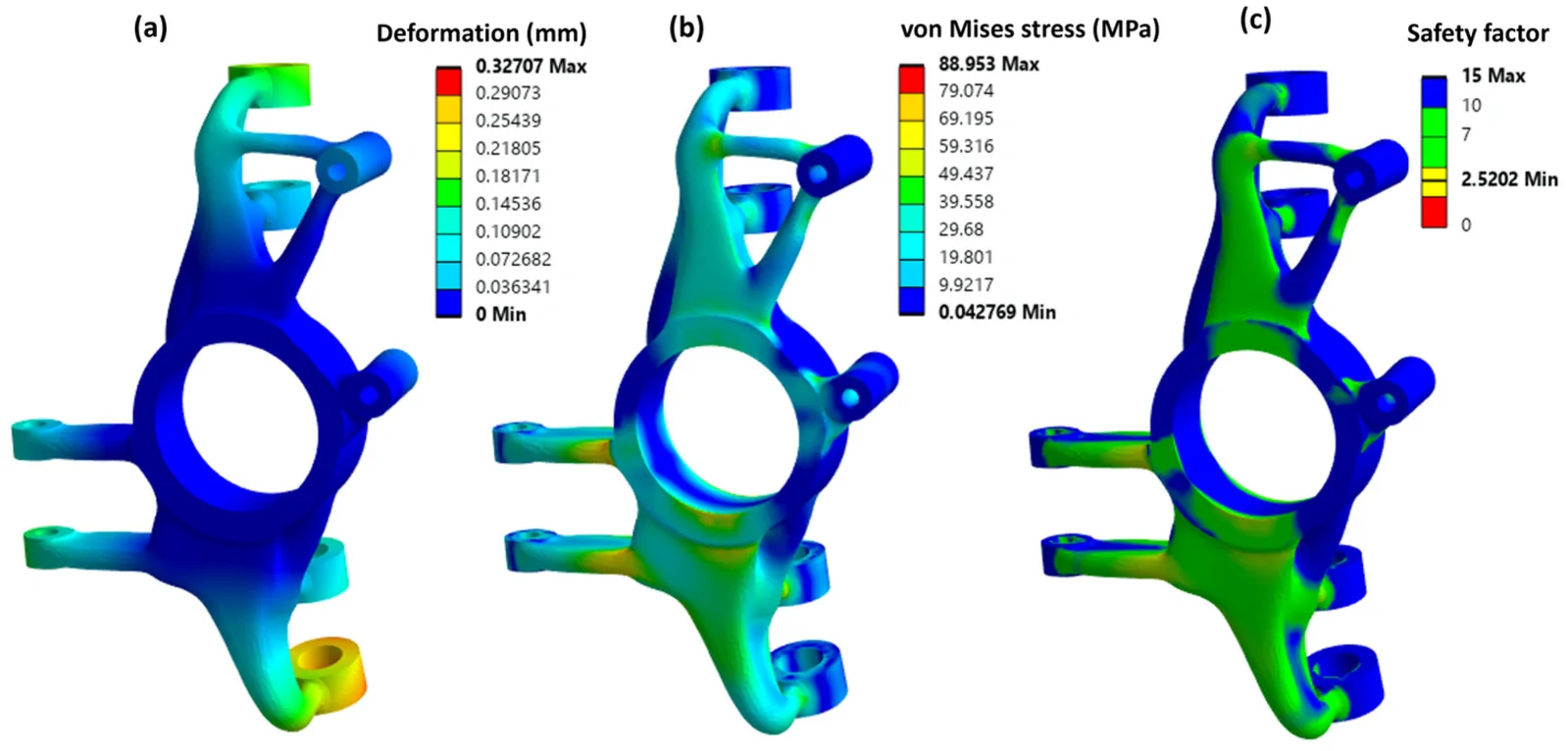
Finite element results for the optimized Formula SAE steering knuckle under the evaluated combined static loading conditions: (**a**) deformation (mm), (**b**) von Mises stress (MPa), and (**c**) safety factor.

**Figure 14:**
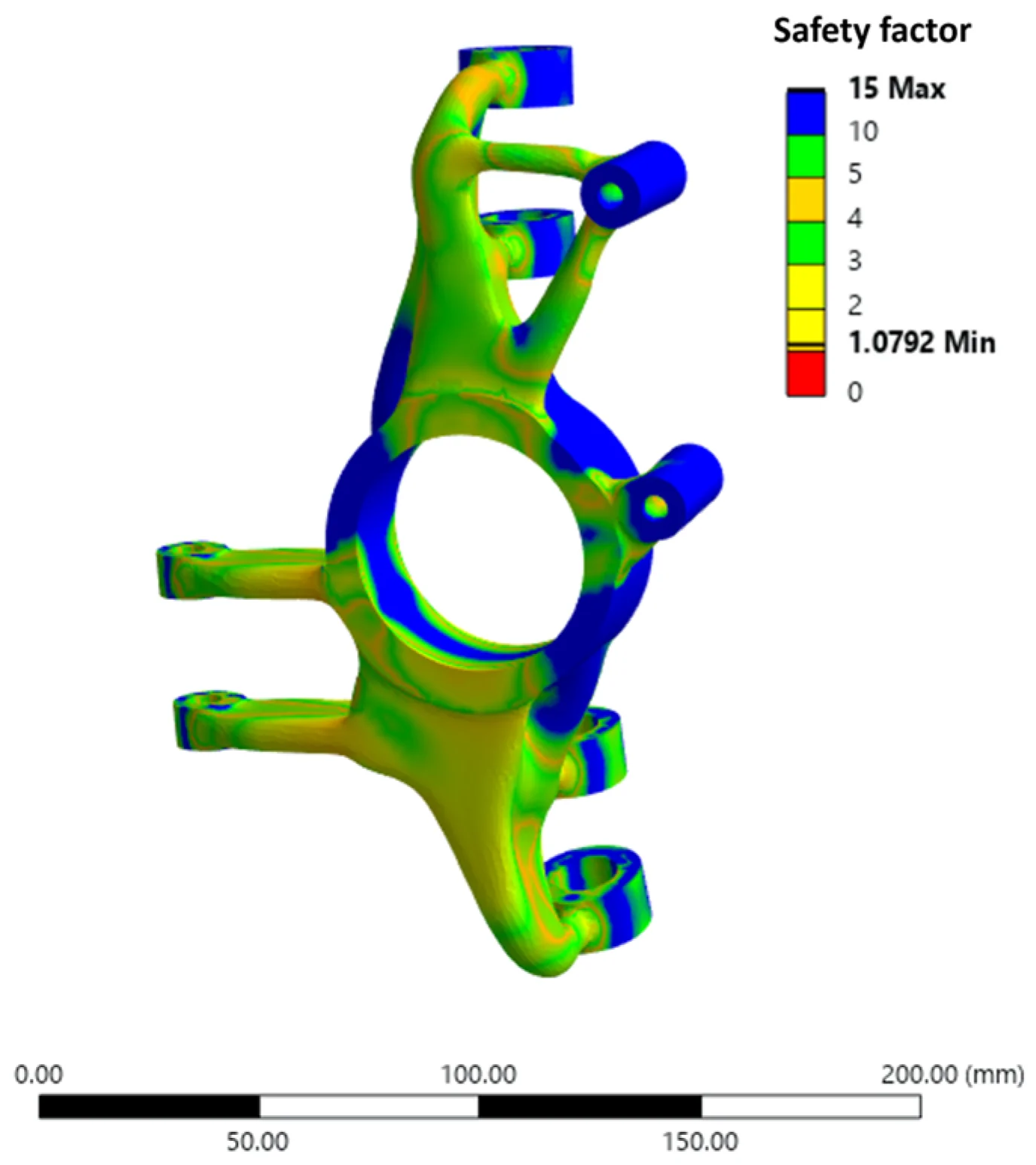
Preliminary fatigue safety-factor distribution obtained using ANSYS Mechanical under fully reversed loading (R = −1), zero mean stress, and an experimental run-out reference of 96 MPa at 10^7^ cycles.

**Table 1:** Processing parameters used for the fabrication of AlSi10Mg and AlSi10Mg/SiC specimens.

Parameter	AlSi10Mg	AlSi10Mg/SiC
Laser power, W	400	310
Scan speed, mm/s	1800	1000
Hatch spacing, mm	0.13	0.10
Layer thickness, μm	30	30
Build orientation, °	0	0
Laser rotation angle, °	67	67
Substrate temperature, °C	200	200
Laser focus, mm	0	0

**Table 2:** Representative loading conditions applied to the finite element model of the steering knuckle.

Force	Value (N)
Wishbone left	1000
Upper wishbone upper right	1000
Tie rod steering	2300
Lower wishbone left	1500
Lower wishbone right	1500
Axial force	6000
Radial force	1500
Braking force	2000

**Table 3:** Mesh independence study of optimized design.

Number of Elements	Von Mises Stress (MPa)
2600	51.6
4000	55.0
5500	61.0
10,000	68.5
28,500	82.5
42,000	88.0
65,000	88.9

**Table 4:** Summary of tensile mechanical properties of PBF-LB/M-fabricated AlSi10Mg and AlSi10Mg/SiC specimens (mean ± standard deviation).

Material	Samples	UTS (MPa)	Yield Strength (MPa)	Young’s Modulus (GPa)	Elongation at Failure (%)
AlSi10Mg	4	362.38 ± 4.61	224.18 ± 1.51	60.68 ± 1.94	4.62 ± 0.31
AlSi10Mg/SiC	5	219.79 ± 7.86	193.50 ± 5.65	52.47 ± 5.18	0.86 ± 0.23

**Table 5:** Absorbed energy obtained from Charpy impact testing of PBF-LB/M-fabricated AlSi10Mg/SiC specimens.

Specimen	Absorbed Energy (J)	Absorbed Energy (ft·lbf)
P1	4.0	2.95
P2	5.0	3.69
P3	4.0	2.95
P4	4.0	2.95
P5	3.0	2.21
P6	4.0	2.95
P7	3.0	2.21
P8	3.0	2.21

**Table 6:** Comparison of mass and structural response of the baseline and optimized Formula SAE steering knuckle under the evaluated static loading conditions.

Parameter	Baseline Design	Optimized Design	Change
Mass (kg)	1.13	0.62	−45.1%
Maximum von Mises stress (MPa)	62.57	88.95	+42.2%
Maximum displacement (mm)	0.11	0.32	+190.9%
Minimum safety factor	3.58	2.52	−29.6%

## Data Availability

The original contributions presented in this study are included in the article. Further inquiries can be directed to the corresponding authors.
